# Droppings From Captive *Coturnix coturnix* (Galliformes: Phasianidae) as a Fly Breeding Resource

**DOI:** 10.1093/jisesa/ieu012

**Published:** 2014-01-01

**Authors:** M. Battán Horenstein, I. Lynch-Ianniello, B. de Dio, R. M. Gleiser

**Affiliations:** ^1^ Instituto de Diversidad y Ecologιa Animal (CONICET-UNC). Av. Velez Sarsfield 299, CP 5000, Córdoba, Argentina; ^2^ Facultad de Ciencias Exactas, Físicas y Naturales, Córdoba, Argentina; ^4^ CREAN-IMBIV (CONICET-UNC). Av. Valparaiso s/n - C.C. 509 - C.P. 5000 Córdoba, Argentina

**Keywords:** dung, Calliphoridae, Muscidae, poultry

## Abstract

The aim of this study was to describe the fauna of flies associated with captive *Coturnix coturnix* (L.) (Galliformes: Phasianidae) droppings. Samples of 150 g of quail droppings were exposed in the quail house for 48 h in plastic containers to promote eventual access of flies, and then placed in emergence traps. The number of adults and species emerging was recorded daily. This procedure was carried out in spring 2008 and spring and autumn 2009. In total, 2,138 adults belonging to Muscidae, Calliphoridae, Piophilidae, Phoridae, Fanniidae, and Milichiidae families were collected. The most numerous family was Muscidae (representing >82% of the total specimens), with *Musca domestica* L. being the most abundant species followed by *Ophyra aenescens* (Wiedemann) (both Diptera: Muscidae). Quail breeding should include adequate droppings management policies to avoid potential sanitary issues related to fly production.

Integrated poultry production techniques allow increased egg or meat production in small spaces. In these management systems involving high densities of birds, flies have become a common pest, abundant and difficult to handle in poultry establishments, and fly density is proportional to the accumulation of wet bird droppings ( Axtell and Arends 1990 ). Although the removal and drying or composting of poultry manure is a recommended procedure to decrease the production of flies, in itself it is not enough to prevent the development of these insects ( Moon et al *.* 2001 ). Flies, mainly house flies, blow flies, and flesh flies, are important from a sanitary point of view as vectors of pathogen, and are known to carry human intestinal bacteria ( Béjar et al. 2006 ), parasites, human protozoa ( Cárdenas and Martínez 2004 ), and animal metazoan parasites ( Keiding 1986 , Graczyk et al *.* 1999 , Barnard 2003 ). Flies not only contribute to the spread of diseases as the primary health factor, but they also may exert stress on the birds and increase levels of ammonia (due to the activity of the larval stages of the fly in the poultry bed manure) as secondary factors. In addition, defects of the eggshells of birds, e.g., due to fly feces stains, can generate substantial economic losses ( Shane 2008 ).

Breeding of quail is an emerging economic activity as an alternative source of meat and eggs, and small household production is becoming more widespread. To our best knowledge, there are no reports of fly species developing in quail droppings, especially in South America. In Argentina, fly fauna associated with poultry farms and poultry houses has been studied mainly focusing on *Musca domestica* L. (Diptera: Muscidae) and biological control issues ( Crespo et al *.* 1998 , Lecuona et al. 2007 , Di Iorio and Turienzo 2011 ). The knowledge about dung and manure flies is important for the implementation of control programs of pests that breed in poultry productions. Because not all manure sources are equally productive or adequate for fly development, the aim of this study was to describe the fauna of flies associated with captive Japanese quail [ *Coturnix coturnix* (L.)] droppings and determine whether they are used as a breeding and development substrate by these species.

## Materials and Methods

### 

#### Sampling Procedure

Droppings were collected from Japanese quail reared following similar methods to those described elsewhere ( Marin and Satterlee 2003 , Kembro et al. 2008 ). Birds were housed in groups of one male and three females in 20 by 45 by 25 cm (length by width by height) cages within six-tier cage batteries (each battery comprising 24 laying cages). Birds were fed a laying diet (Marcelo E. Hoffman e Hijos S.A., Entre Ríos, Argentina) containing corn meal, soybean meal, wheat shorts, sunflower meal, limestone, sodium chloride, dicalcium phosphate, vitamins, and minerals with 21.5% crude protein and 2,750 kcal ME/kg, with feed and water provided ad libitum. The daily photostimulatory cycle was 16:8 (L:D) h with a light intensity of ∼280 lx during the lighted portion of the day and lights-on occurring at 0600 hours daily. Cage trays were cleaned (0830 hours), and 24 h later, droppings were collected, and samples of 150 g were placed in plastic containers following the general procedure described by Goulson et al *.* (1999) . Each plastic container held droppings from one quail cage. The samples were taken from seven, six, and five cages in spring 2008, spring 2009, and fall 2009, respectively (equal to total containers per season). The plastic containers with the quail droppings were exposed for 48 h in the rearing quail room to promote eventual access of flies, and then placed in emergence traps. A trap consisted of a larger dark-walled plastic container, with a layer of sand covering its bottom for pupae to complete development, and connected through a funnel to a removable adult collection bottle.

The emergence traps were monitored daily for 40 d to register the emergence of adult flies. Emerging adults were removed and counted daily, stored in 90% ethanol, and determined at a specific level (when possible) based on morphological characters ( McAlpine et al. 1981a , 1987 ; Carvalho 2002 ; Mariluis 2002 ). During the study, the temperature inside the breeding room was 25 ± 2°C. The following parameters were estimated: total number of flies as a whole and per species, species richness and diversity (Shannon-W and Simpson), and average number of days to adult emergence. Diversity indexes were estimated with InfoStat (2002) using 250 bootstrap cycles and 0.95 confidences.

#### Statistical Analysis

Adult fly emergence (total and per species), richness, and diversity parameters were subjected to an analysis of variance (ANOVA) that examined the main effects of season. Data on *Coproica* Rondani (Diptera: Sphaeroceridae) abundance were transformed to ranks before ANOVA testing ( Shirley 1987 ) to fit better the assumptions of the test. Least significant difference Fisher’s test was used for post hoc analyses. A *P* value of <0.05 was considered to represent significant differences. A principal components analysis (PCA) was used to explore the association of species presence and abundance with seasons.

## Results

In all, 2,138 adults belonging to Muscidae, Calliphoridae, Piophilidae, Sphaeroceridae, Fanniidae, and Milichiidae families were collected. The composition and abundance of flies observed each season are listed in Table 1 . The most numerous family was Muscidae (>82% of emerging specimens), and *M. domestica* was the most abundant species followed by *Ophyra aenescens* (Wiedemann) (Diptera: Muscidae). The second most abundant family was Sphaeroceridae, represented mainly by *Coproica* spp. The Calliphoridae family was very scarcely represented both numerically as in diversity of species. One species, *Lucilia sericata* Meigen (Diptera: Calliphoridae), was collected only in spring 2009.

**Table 1.** Composition, frequency, and percentage of adult flies emerging from Japanese quail droppings collected in spring 2008 and 2009, and fall 2009

	Spring 2008	Spring 2009	Fall 2009	Percentage
Muscidae				
*M. domestica*	108 (15.4 ± 12.7 ^a^ )	467 (77.8 ± 28.2 ^b^ )	522 (104.4 ± 13.8 ^b^ )	51.31
*O. aenenscens*	91 (13 ± 8.1 ^a^ )	207 (34.5 ± 14.3 ^a^ )	201 (40.2 ± 11 ^a^ )	23.34
*Musci. stabulans*	82 (11.7 ± 4.1 ^a^ )	85 (14.2 ± 7.6 ^a^ )	1 (0.2 ± 0.2 ^a^ )	7.86
Sphaeroceridae				
*Coproica*	4 (0.6 ± 0.4 ^a^ )	77 (12.8 ± 11.5 ^a^ )	167 (33.4 ± 19.9 ^b^ )	11.6
Calliphoridae				
*L. sericata*^1^	0	21	0	0.98
Piophilidae				
*P. casei*^1^	0	0	9	0.42
Fanniidae				
*Fannia*^1^	0	2	0	0.09
Milichiidae				
Undetermined ^1^	93	0	0	4.35

For individual species, different letters (a,b) indicate significant differences in adult emergence between seasons. Mean ± SE values are given in parenthesis.

^1^ No statistical assessments were carried out due to low number of flies and positive containers.

**Fig. 1.**
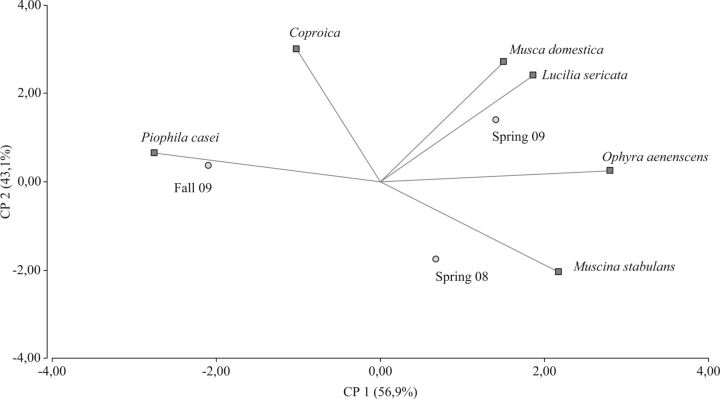
PCA showing association between species abundance and seasons.

An ANOVA of the total fly abundance showed significant effects of season ( *F*_2,15_ = 6.08; *P* < 0.05), and fly emergence was significantly lower during spring 2008 compared with spring or fall 2009 ( Table 2 ). No significant differences were detected in species richness ( *F*_2,15_ = 1.42; *P* = 0.27) or diversity between seasons ( *F*_2,15_ = 2.35; *P* = 0.13; *F*_2,15_ = 1.82; *P* = 0.20; and *F*_2,15_ = 1.45; *P* = 0.26, for Shannon-W, C.D. Simpson, and Simpson indexes, respectively) ( Table 2 ). A PCA shows the close relationship between species presence or abundance and season. Both springs are clearly separated from fall regarding species composition ( Fig. 1 ). *M.domestica* showed significant season effects ( *F*_2,15_ = 5.6; *P* < 0.05), and presence and abundance were lower during spring 2008. Emergence of *Coproica* spp. showed a similar pattern and was higher during the fall compared with both springs ( *F*_2,15_ = 7.9; *P* < 0.01). No significant differences were detected between seasons in the abundance of *Muscina stabulans* (F.) (Diptera: Muscidae) ( *F*_2,15_ = 1.84; *P* = 0.19) and *O.aenescens* ( *F*_2,15_ = 1.71; *P* = 0.21). No statistical assessments were carried out for the remaining species due to a low number of flies emerging from few containers ( Table 1 ).

**Table 2.** Total abundance, richness, and diversity of adult flies emerging from Japanese quail droppings collected in spring 2008 and 2009, and fall 2009

Parameter	Spring 2008	Spring 2009	Fall 2009
Abundance	54 ± 15.9 ^a^	180 ± 34.6 ^b^	143 ± 29.4 ^b^
Richness	2.6 ± 0.2 ^a^	3.33 ± 0.6 ^a^	3.4 ± 0.2 ^a^
Shannon-W	0.5 ± 0.1 ^a^	0.8 ± 0.1 ^a^	0.8 ± 0.2 ^a^
Simpson	0.7 ± 0.1 ^a^	0.5 ± 0.1 ^a^	0.5 ± 0.1 ^a^
C.D. Simpson	0.7 ± 0.1 ^a^	0.6 ± 0.1 ^a^	0.5 ± 0.1 ^a^

Mean ± SE values are reported. For each parameter, different letters (a,b) indicate significant differences in adult emergence between seasons ( *P* < 0.05).

The average number of days to adult emergence was estimated for the most abundant species collected in all seasons ( Table 3 ). *O. aenescens* adult emergence times were significantly shorter in both springs (15.8 ± 1.3 in spring 2008; 17.1 ± 0.1 in spring 2009) compared with fall (32.7 ± 2.0 d) ( *F*_2,11_ = 12.37; *P* < 0.01; data transformed to ranks). *M.domestica* showed a similar pattern, although differences between spring (13.2 ± 0.0 and 12.3 ± 0.8 for spring 2008 and 2009, respectively) and fall (16.2 ± 0.6 d) were smaller ( *F*_2,9_ = 8.67; *P* < 0.01). *Musci.stabulans* emerged in significantly fewer days in spring 2009 (17.7 ± 1.0) compared with spring 2008 (21.1 ± 1.0 d) and fall (25 ± 0.0 d) ( *F*_2,8_ = 5.44; *P* < 0.05).

**Table 3.** Average number of days to adult emergence of the three most abundant flies emerging from Japanese quail droppings collected in spring 2008 and 2009, and fall 2009

Species	Season		
	Spring 2008	Spring 2009	Fall 2009
*O. aenenscens*	15.84 ± 1.33 ^a^	17.11 ± 0.11 ^a^	32.73 ± 2.03 ^b^
*Musci. stabulans*	21.13 ± 1.03 ^a^	17.73 ± 1.01 ^b^	25.00 ± 0.00 ^a^
*M. domestica*	13.18 ± 0.01 ^a^	12.34 ± 0.81 ^a^	16.19 ± 0.59 ^b^

Mean ± SE values are reported. For individual species, different letters (a,b) indicate significant differences in average days to adult emergence ( *P* < 0.05).

No significant correlations were detected between the numbers of flies emerging of the three most frequent species ( Table 4 ).

**Table 4.** Correlation (Spearman correlation coefficient) of *O* . *aenescens, M* . *domestica,* and *Musci* . *stabulans* abundance

Species	Season		
	Spring 2008	Spring 2009	Fall 2008
*O. aenenscens* and *M. domestica*	0.32, *P* = 0.43	0.03, *P* = 0.95	−0.58, *P * = 0.25
*O* . *aenenscens* and *Musci* . *Stabulans*	−0.09, *P * = 0.88	0.60, *P * = 0.18	0.78, *P * = 0.12
*M* . *domestica* and *Musci* . *Stabulans*	−0.06, *P * = 0.83	−0.49, *P * = 0.28	−0.25, *P * = 0.62

*P* values, probability values α = 0.05.

## Discussion

Poultry manure and exposed wet feed are ideal feeding and breeding materials for several fly species; thus, fly production is a problem associated with poultry housing. A recent bibliographic review indicates that in neotropical regions over 14 Diptera species and another 12 flies determined to the genus or family level are associated with poultry housing [ *Gallus gallus* (L.), Aves: Phasianidae] ( Di Iorio and Turienzo 2011 ). However, in Argentina, only three species besides *M. domestica* have been reported in this environment: *Philornis angustifrons* (L.) (Diptera: Muscidae), *Fannia albitarsis* Stein (Diptera: Fanniidae), and *Hermetia illucens* (L.) (Diptera: Stratiomyidae). This is the first report of flies developing in quail droppings, where nine different flies comprising six families were detected; five of them were identified to the species level [ *M. domestica, Musci. stabulans, O. aenescens, L. sericata,* and *Piophila casei* (L *.* ) (Diptera: Piophilidae)], two to the genus level ( *Coproica* and *Fannia* ), and one identified to the family level (Milichiidae).

In this study, we found the same families reported by other authors in association with dung or feces. The species reported here were also observed in different manure sources (from cattle) ( Mendes and Linhares 2002 , Chong Chin et al *.* 2010 , Floate 2011 ). The low abundance of *L.sericata* , *Fannia* sp., and *P. casei* would be the result of breeding preference by these species for other kinds of decomposing resource, such as corpses of dead animals, cheese, or older dung ( Mendes and Linhares 2002 , Battán Horenstein et al *.* 2010 ).

Muscidae flies present a wide variety of feeding habits. Depending on species, adults can prey on other insects, or suck blood or sugary substances, among other feed sources, whereas the larvae can be reared on feces, other organic waste, fruit, fungi, plants, and rotting animal carcasses ( Borror et al. 1989 ). *M.domestica* is a cosmopolitan species ( Smith 1986 ) adapted to various substrates, including carcasses ( Oliveira et al. 2002 ). This species is a common filth fly found in broiler-breeder houses ( Axtell and Arends 1990 ) and is considered the main fly species of relevance in poultry houses in Argentina ( Crespo et al. 1998 ). Consistently, in this study, *M. domestica* was the most frequent species of the Muscidae family observed throughout the two seasons ( Table 1 ), indicating that quail droppings are also a suitable environment for the development of this species.

*O* *.* *aenescens* was the second most frequent species emerging from the traps. It is native to the American continent but currently is found in different parts of the world ( D'Almeida et al. 1999 ). According to Bohart and Gressit (1951), it takes advantage of a wide variety of feed substrates, including feces, carcasses, and decomposing plants. Anderson and Poorbaugh (1964) report predator behavior in several species of the genus *Ophyra* during the larval stage. *O. aenescens* larvae, during their development, are facultative larval predators on *M.domestica* larvae ( Hogsette and Jacobs 1999 ). This species is used in the biological control of *M. domestica* in poultry and pig farms in the United States and Europe ( Farkas et al. 1998 , Hogsette and Jacobs 1999 ). However, we did not find any significant correlations between the abundance of *O. aenescens* and numbers of other flies emerging (potential prey). Thus, in this study, it was considered a coprophagous community component.

*Musci* *.* *stabulans* is a widely distributed species ( Smith 1986 ), most frequently found in the Neotropics ( Carvalho 2002 ). Adult *Musci.stabulans* are usually collected in open spaces, around stables, chicken houses, or decomposing organic material ( Smith 1986 ). Larvae of this species may develop on decomposing fungi, fruit, broken eggs, feces, and animal carcasses. They are necrophagous in their early larval stages and may become predatory in later stages ( Smith 1986 ). Even though it was collected in low numbers from the quail droppings samples, this species is widely reported as part of the sarcosaprophagous fauna ( Reed 1958 , Tantawi et al. 1996 ).

Coprophagy is a widespread larval feeding strategy in Spheaeroceridae ( Buck 1997 ). In this study, adults from the genus *Coproica* were collected in spring and fall 2009. Several species of this genus have been reported as common fauna associated with feces ( Buck 1997 ). *Coproica* was mentioned by Battán Horenstein et al. ( 2010 ) as a component of carcasses decomposing fauna in a rural area of Córdoba (Argentina). However, as far as we know, this study is the first that mentioned *Coproica* genus in relation with bird droppings as breeding resource in Argentina.

The Milichiidae family is rather diverse. The larvae are generally saprophagous and live in decaying plants, but several are coprophagous or even necrophagous ( McAlpine et al. 1981a , 1987 ). Milichiidae were recorded only in spring 2008 in two containers, and 99% of the flies emerged from only one of them. It is important to point out that this study is the first to report the emergence of adult Milichiidae flies from quail droppings or manure in the Neotropics.

During the exposition period (48 h), quail droppings were colonized by several dipteran species that lay eggs and bred, resulting in the emergence of adults. Thus, quail breeding should include adequate droppings management policies to avoid potential sanitary issues related to fly production. The knowledge of the entity of the species that are present in poultry houses or farms is the first step toward pest control management. Data on the specific composition of Diptera present in quail farms can contribute to the establishment of appropriate management policies that may include both cultural management of droppings and the use of biological control (e.g., using specific hymenopteran parasitoid species).
